# Synthesis of novel isoxazole–carboxamide derivatives as promising agents for melanoma and targeted nano-emulgel conjugate for improved cellular permeability

**DOI:** 10.1186/s13065-022-00839-5

**Published:** 2022-06-24

**Authors:** Mohammed Hawash, Nidal Jaradat, Ahmad M. Eid, Ahmad Abubaker, Ola Mufleh, Qusay Al-Hroub, Shorooq Sobuh

**Affiliations:** 1 Department of Pharmacy, Faculty of Medicine and Health Sciences, An-Najah National University, Nablus, 00970 Palestine; 2 Department of Biomedical Sciences, Faculty of Medicine and Health Sciences, An-Najah National University, Nablus, 00970 Palestine

**Keywords:** Isoxazole, Anticancer, B16F1, LX-2, Nano, Doxorubicin

## Abstract

**Background:**

Cancer is one of the most dangerous and widespread diseases in the world today and it has risen to the position of the leading cause of death around the globe in the last few decades. Due to the inherent resistance of many types of cancer to conventional radiotherapy and chemotherapy, it is vital to develop innovative anticancer medications. Recently, a strategy based on nanotechnology has been used to improve the effectiveness of both old and new cancer drugs.

**Objectives:**

The present study aimed to design and synthesize a series of phenyl-isoxazole–Carboxamide derivatives, evaluate their anticancer properties, and improve the permeability of potent compounds into cancer cells by using a nano-emulgel strategy.

**Methods:**

The coupling reaction of aniline derivatives and isoxazole–Carboxylic acid was used to synthesize a series of isoxazole–Carboxamide derivatives. IR, HRMS, 1H-NMR, and 13C-NMR spectroscopy techniques, characterized all the synthesized compounds. The *in-vitro* cytotoxic evaluation was performed by using the MTS assay against seven cancer cell lines, including hepatocellular carcinoma (Hep3B and HepG2), cervical adenocarcinoma (HeLa), breast carcinoma (MCF-7), melanoma (B16F1), colorectal adenocarcinoma (Caco-2), and colon adenocarcinoma (Colo205), as well as human hepatic stellate (LX-2) in addition to the normal cell line (Hek293T). A nano-emulgel was developed for the most potent compound, using a self-emulsifying technique.

**Results:**

All synthesized compounds were found to have potent to moderate activities against B16F1, Colo205, and HepG2 cancer cell lines. The results revealed that the **2a** compound has broad spectrum activity against B16F1, Colo205, HepG2, and HeLa cancer cell lines with an IC_50_ range of 7.55–40.85 µM. Moreover, compound **2e** was the most active compound against B16F1 with an IC_50_ of 0.079 µM compared with Dox (IC_50_ = 0.056 µM). Nanoemulgel was used to increase the potency of the **2e** molecule against this cancer cell line, and the IC_50_ was reduced to 0.039 µM. The antifibrotic activities were investigated against the LX-2 cell line, and it was found that our synthesized molecules showed better antifibrotic activities at 1 µM than 5-FU, and the cell viability values were 67 and 95%, respectively.

**Conclusion:**

This study suggests that a **2e** nano-formalized compound is a potential and promising anti-melanoma agent.

**Supplementary Information:**

The online version contains supplementary material available at 10.1186/s13065-022-00839-5.

## Background

Cancer is a generic word that indicates a large group of about 100 diseases. It can affect any organ in the body and usually occurs when a cell breaks the cell-division restrictions, causing an abnormal proliferation and growth of the cells [1]. In addition to the genetic factor, there are plenty of environmental factors that contribute to the development of different types of cancer, such as high consumption of tobacco and alcohol, a low level of physical activity, exposure to radiation, and viral infection [2–4].

Up until now, cancer has been one of the most lethal common diseases and is the second leading cause of death worldwide [5, 6]. In 2020, 19.3 million new cases were estimated to have cancer, with about 10.0 million deaths [7]. According to recent World Health Organization (WHO) posts, the percentage of common cancer types was 2.26 million in the breast, 2.21 million in the lung, 1.93 million in the rectum and colon, 1.2 million in the skin, and about 1 million in the liver [1, 8].

Skin cancer is divided into two types: melanoma is the sixth most common cancer in women and the fifth most common cancer in men [9], and is considered one of the most common types in the United States, where one in every five Americans will have cancer by the age of seventy. Skin color is considered one of the risk factors for melanoma, which is 20 times more prevalent in white people than in black people [10–12]. Early diagnosis of melanoma is a critical point in treatment because it decreases long-term and short-term mortality and morbidity. There is a series of pathways to diagnosis, which first occurs visually by using dermoscopy, then taking a biopsy, and making a histopathological assessment. The melanoma has special architectural features that are different from those in a normal cell, which are: a gathering of growth, asymmetrical in shape, and outstanding nucleoli with a thick and unequal nuclear membrane [9, 13, 14]. A lot of strategies are used for treatment, like surgical removal, immunotherapy, and chemotherapy. But the type of treatment used depends on the stage of the tumor [9].

Liver fibrosis is considered the major consequence of pathological hepatic diseases and clinically represents the major complications of the last stage of hepatic disease. As liver fibrosis advances, a large amount of extracellular matrix (ECM) like collagen is produced and accumulated, which leads to hepatic dysfunction and cirrhosis. The main cellular origin of the extracellular matrix is the hepatic stellate cells (HSC). HSC, in its quiescent state, is considered the main site for the storage of vitamin A, which is necessary for the homeostasis regulation of retinoic acid. When a hepatic injury occurs, the HSC will transform into a myofibroblast-like phenotype. This change is associated with liver fibrosis, which will produce a lot of growth factors, and cytokines, and the ESM will be remodeled. The LX-2 cell line is an unbounded source of human HSC. It has activated HSC features like retinoid metabolism, cytokine signaling, fibrogenesis, and neuronal gene expression [15]. The anti-fibrotic agents usually induce apoptosis via various regulatory pathways, including the mitochondrial-induced apoptosis members of the bax and bcl-2 families, and phosphorylated mitogen-activated protein kinases, containing c-Jun N-terminal, extracellular (EC) signal-regulated kinases and p38 protein [15, 16].

Chemotherapy is still an essential therapy in most cancer types, whether it is used alone or in conjunction with another strategy of treatment [6, 17]. In recent decades, a lot of studies have aimed to make novel chemotherapeutic drugs, by making modifications to existing ones [18, 19], or isolating compounds from plants [20].

**Fig. 1:**
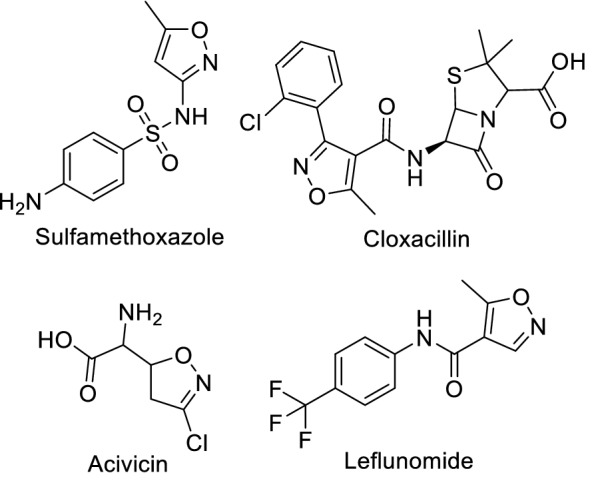
Isoxazole–Containing drugs with various pharmacological activities

A lot of heterocyclic compounds have anticancer activity, such as isoxazole [18, 21] and pyrazole [22–25]. Recently, numerous studies have been conducted on heterocycles such as isoxazole, pyrazole, and thiazole derivatives as pharmacologically active agents [26–32]. Heterocyclic isoxazole is a 5-membered ring containing three carbon atoms, a nitrogen atom, and an oxygen atom adjacent to each other [33, 34]. They found that it had anticancer [35–37], antiviral for HIV [38], antimicrobial [39, 40], anti-inflammatory [41, 42], analgesic [39], hypoglycemic [43], and antioxidant activity [33]. Some of these compounds have been approved and become available on the market, like Sulfamethoxazole (Fig. 1) [44] and Cloxacillin (Fig. 1), which have antibacterial activity [45], and Acivicin (Fig. 1), which has anticancer activity [46], A lot of the synthesized compounds consisted of an aryl group with halogen substitute and a carboxamide linker that bound it with isoxazole group found to have anti-tumor activity, and one of these compounds was Leflunomide (Fig. 1) which was approved for the treatment of rheumatoid arthritis. Recently, this drug was shown to be antiproliferative against bladder cancer cell lines and can induce cell cycle arrest at the S phase of the cell cycle [47].

To reduce the undesirable side effects of chemotherapy, a new branch of alternative therapeutic approach to more specific targets of cancer cells without harming normal cells has been developed. A nanotechnology strategy has been used recently to enhance the effects of both traditional and new pharmacological anticancer treatments. The latest development in the drug delivery field is using nanoparticles (NPs) in a more specific way to reach tumor cells. Due to its small size (1 to 100 nm), it accumulates in tumor cells [48].

In our previous work, different series of isoxazole derivatives were synthesized and evaluated as anticancer agents, and generally, isoxazole–Carboxamide derivatives exhibited moderate to potent antiproliferative activities against panel cancer cell lines with low values of IC_50_s [18, 49]. The current study aims to synthesize a novel 5-methyl-3-phenylisoxazole-4–Carboxamide with several substituents and evaluate its antiproliferative activities against various kinds of cancer cell lines, including B16-F1, Colo205, HepG2, CaCo-2, HeLa, Hep3B, MCF-7, and normal hepatic LX-2 and kidney Hek293t cells. In addition to creating a nanoemulgel to boost the potency of the most active compound on B16-F1.

## Methods

### Chemistry

All used chemical reagents and starting materials were ordered from Alfa Aesar (UK) and Sigma-Aldrich (Germany). All used cancer, and normal cell lines were ordered from ATCC. The SMP-II Digital Melting Point Apparatus was used without correcting to find the melting points (M.P.) of all final compounds. ^1^H-NMR and ^13^C-NMR spectra were recorded by using DMSO-d_6_ as the solvent and were conducted by the Bruker 500 J MHz-Avance III High-Performance Digital FT-NMR spectrometer at the Chemistry department, Science Faculty, in the University of Jordan, Amman-Jordan. However, the chemical shifts were recorded accordingly as δ (ppm). The used mass instrument was the High-resolution mass spectrometer (HRMS) at the Phramceuteical chemistry department, Pharmacy Faculty in Gazi University, Ankara-Turkey, this instrument uses the Waters LCT Premier XE Mass Spectrometer which is coupled to an AQUITY Ultra Performance Liquid Chromatography (UP-LC) system (Waters Corporation, Milford, MA, USA) [50].

### The general synthesis procedure of phenyl-isoxazole–Carboxamide derivatives (2a-2f)

The 5-(methyl)-3-phenyl-1,2-isoxazole-4–Carboxylic acid (compound **1**) (609.60 mg, 3 mmol) was dissolved in 20 ml of dichloromethane (DCM), followed by the addition of di methylamino-pyridine (DMAP; 73.30 mg, 0.6 mmol), and N′-ethyl carbodiimide hydrochloride (EDC; 632.61 mg, 3.30 mmol). Then the mixture was stirred under argon inert gas at room temperature for 30 min, and the aniline derivatives (3.2 mmol) were added. The reactions were monitored by thin-layer chromatography (TLC). After that, the solvent was removed under vacuum pressure and dissolved again in DCM, then extracted with HCl (2 N) to remove any excess aniline derivatives. The organic layer was dried over Na_2_SO_4_ and evaporated under reduced pressure. The obtained final products were purified by flash chromatography using the convenient solvent systems (DCM: ethyl acetate and/or n-hexane: ethyl acetate) and/or then purified by recrystallization utilizing the convenient solvent system [18].

### N-(4–Chloro-2,5-dimethoxyphenyl)-5-methyl-3-phenylisoxazole-4–Carboxamide (2a)

This product was purified by column chromatography using an n-hexane: ethyl acetate (3:2) solvent system, TLC Retention factor of 0.644. Solid product, M.P. 193–195 °C, Yield 67%, 749.35 mg; ESI–MS: 373.0894 (100), 375.0880 (33), [M + H]^+^ calcd. 373.0894, found. 373.0955 For C_19_H_17_ClN_2_O_4_. IR (FTIR/FTNIR-ATR): 1666.46 cm^−1^ amide carbonyl (C=O). ^1^H NMR (DMSO-d_6_) δ: 9.20 (1H, s, NH), 7.88 (1H, s, Ar–H), 7.70–7.56 (5H, m, Ar–H), 7.14 (1H, s, Ar–H), 3.79 (3H, s, –OCH_3_), 3.64 (3H, s, –OCH_3_), 2.67 (3H, s, –CH_3_) ppm. ^13^C NMR (DMSO-d_6_) δ: 172.41, 160.65, 148.57, 144.75, 130.68, 129.39, 128.86, 128.30, 126.58, 116.74, 113.78, 112.79, 107.94, 56.99, 56.94, 12.83 ppm.

### 5-Methyl-3-phenyl-N-(3-(trifluoromethyl) phenyl) isoxazole-4–Carboxamide (2b)

This product was purified by column chromatography using DCM: ethyl acetate solvent system (4:1). The TLC retention factor was 0.95. Solid product, M.P. 148–150 °C, Yield 59%, 614.37 mg; ESI–MS: 347.0995 (100), 348.1052 (20), [M + H]^+^ calcd. 347.0995, found 347.1007 for C_18_H_13_F_3_N_2_O_2_. IR (FTIR/FTNIR-ATR): 1680.73 cm^−1^ amide carbonyl (C=O). ^1^H NMR (DMSO-d_6_) δ: 10.78 (1H, s, NH), 8.14 (1H, s, Ar–H), 7.83–7.50 (8H, s, Ar–H), 2.63 (3H, s, –CH_3_) ppm. ^13^C NMR (DMSO-d_6_) δ ppm: 170.88 (C=O), 160.91, 160.78, 139.82, 130.61, 130.58, 130.14, 129.33, 128.39, 128.29, 123.77, 120.89, 120.88, 116.28, 116.25, 113.40, 12.46 (–CH_3_) ppm.

### N-(4-(2-Methoxyphenoxy) phenyl)-5-methyl-3-phenylisoxazole-4–Carboxamide (2c)

This product was purified by column chromatography using an *n*-hexane: ethyl acetate solvent system (3:2). The TLC retention factor was 0.49. Solid product, M.P. 179–180 °C, Yield 71%, 854.45 mg; ESI–MS: 401.1497 (100), 402.1516 (33), [M + H] + calcd. 401.1497, found. 401.1501, for C_24_H_20_N_2_O_4_. IR (FTIR/FTNIR-ATR): 1677.61 cm^−1^ amide carbonyl (C=O). ^1^H NMR (DMSO-d_6_) δ: 10.40 (1H, s, NH), 7.72 (2H, s, Ar–H), 7.58 (2H, d, *J* = 8.5 Hz, Ar–H), 7.50 (3H, s, Ar–H), 7.17 (2H, t, J = 9.5 Hz, Ar–H), 7.02–6.95 (2H, m, Ar–H), 6.85 (2H, d, J = 8.5 Hz, Ar–H), 3.76 (3H, s, –OCH_3_), 2.59 (3H, s, –CH_3_) ppm. ^13^C NMR (DMSO-d_6_) δ: 170.21 (C=O), 160.64, 160.15, 154.51, 151.67, 144.43, 133.63, 130.56, 129.31, 128.57, 128.19, 125.79, 121.86, 121.61, 121.53, 117.03, 113.84, 113.79, 56.08 (–OCH_3_), 12.36 (–CH_3_) ppm.

### 5-Methyl-N-(4-(methylthio) phenyl)-3-phenylisoxazole-4–Carboxamide (2d)

This product was purified by column chromatography using an n-hexane: ethyl acetate solvent system (3:2). The TLC Retention factor was 0.66. Solid product, M.P. 144–146 °C, Yield 81%, 789.99 mg; ESI–MS: 325.1008 (100), 326.1086 (20), [M + H]^+^ calcd. 325.1008, found. 325.1011, for C_18_H_16_N_2_O_2_S. IR (FTIR/FTNIR-ATR): 1650.32 cm^−1^ amide carbonyl (C=O). ^1^H NMR (DMSO-d_6_) δ: 10.45 (1H, s, NH), 7.70 (2H, s, Ar–H), 7.60 (2H, d, *J* = 7.5 Hz, Ar–H), 7.50 (3H, s, Ar–H), 7.28 (2H, d, J = 8 Hz, Ar–H), 2.59 (3H, s, -SCH_3_), 2.46 (3H, s, –CH_3_) ppm. ^13^C NMR (DMSO-d_6_) δ: 170.35 (C=O), 160.65, 160.30, 136.50, 133.41, 130.58, 129.31, 128.52, 128.20, 127.54, 120.84, 113.75, 15.94 (-SCH_3_), 12.38 (–CH_3_) ppm.

### 5-Methyl-3-phenyl-N-(4-(trifluoromethoxy) phenyl) isoxazole-4–Carboxamide (2e)

This product was purified by column chromatography using an n-hexane: ethyl acetate solvent system (3:2). The TLC Retention factor was 0.69. Solid product, M.P. 158–160 °C, Yield 67.5%, 735.27 mg; ESI–MS: 363.0958 (100), 364.0972 (20), [M + H] + calcd. 363.0958, found. 363.0957, for C_18_H_13_F_3_N_2_O_3_. IR (FTIR/FTNIR-ATR): 1659.55 cm^−1^ amide carbonyl (C=O). ^1^H NMR (DMSO-d_6_) δ: 10.66 (1H, s, NH), 7.76 (2H, d, *J* = 7.5 Hz, Ar–H), 7.70 (2H, s, Ar–H), 7.50 (3H, s, Ar–H), 7.37 (2H, d, J = 8 Hz, Ar–H), 2.60 (3H, s, –CH_3_) ppm. ^13^C NMR (DMSO-d_6_) δ: 170.59 (C=O), 160.69, 160.61, 155.64, 144.57, 138.26, 130.60, 129.32, 128.43, 128.20, 122.20, 121.60, 113.53, 12.40 (–CH_3_) ppm.

### 5-Methyl-3-phenyl-N-(4-(thiophen-2-yl) phenyl) isoxazole-4–Carboxamide (2f)

This product was purified by column chromatography using an n-hexane: ethyl acetate solvent system (3:2). The TLC Retention factor was 0.75. Solid product, M.P. 142–144 °C, Yield 79%, 855.79 mg; ESI–MS: 361.0948 (100), 362.0909 (20), [M + H]^+^ calcd. 361.0948, found. 361.1011, for C_21_H_16_N_2_O_2_S. IR (FTIR/FTNIR-ATR): 1650.24 cm^−1^ amide carbonyl (C=O). ^1^H NMR (DMSO-d_6_) δ: 10.56 (1H, s, NH), 7.71–7.47 (11H, m, Ar–H), 7.13 (1H, s, Ar–H), 2.61 (3H, s, –CH_3_) ppm.^13^C NMR (DMSO-d_6_) δ ppm: 170.46 (C=O), 160.69, 160.41, 143.51, 138.50, 130.60, 130.10, 129.33, 128.92, 128.53, 128.22, 126.37, 125.65, 123.65, 120.60, 113.72, 12.47 (–CH_3_) ppm.

### Potent compound nano-emulgel preparation

The nano-emulgel was developed by combining the potent prepared compound nanoemulsions with Carbopol 940 hydrogel. Therefore, the first step was to prepare mineral oil nanoemulsion formulations using a self-emulsifying technique by mixing oil, surfactant (Tween 80), and co-surfactant (Span 80). Then, the optimum nanoemulsion formulation was chosen based on the droplet size and polydispersity index of the mineral oil in the formulation. According to the study, a ternary phase diagram was constructed using various quantities of mineral oil, Tween 80, and Span 80 to determine the optimal nanoemulsion formulation. Each formulation was weighed and vortexed for 2 min. The nanoemulsion was then self-emulsified in distilled water with mild agitation. The size and distribution of the mineral oil and surfactant emulsion droplets were measured using a NanoBrook Omni 280173 (Brookhaven, New York), which also allowed for the determination of the droplet's polydispersity index (PDI) and diameter. Following that, the optimal formulation was chosen to be loaded with the potent compound. It was then combined with 0.4 percent Carbopol 940 hydrogel to create the potent compound nano-emulgel. The PDI, droplet size, and zeta potential were subsequently measured for the obtained nano-emulgel [51].

### Chemo-informatics parameters of the synthesized compounds

Various websites were used to determine the chemo-informatics characteristic and Lipinski rule of five (RO5) including Molsoft (http://www.molsoft.com/) and Molinspiration (http://www.molinspiration.com)[52].

## Biological methods

### Cell culture and MTS assay

Hepatocellular carcinoma (Hep3B and HepG2), cervical adenocarcinoma (HeLa), breast carcinoma (MCF-7), melanoma (B16F1), colorectal adenocarcinoma (Caco-2), and colon adenocarcinoma (Colo205), as well as human hepatic stellate (LX-2) in addition to the normal cell line (Hek293T), were used as cancer and normal cell lines and were cultured in RPMI-1640 media and supplemented with 10.0% fetal bovine serum, 1.0% l-glutamine and 1.0% Penicillin/Streptomycin antibiotics. After that, the cells matured in a moist atmosphere with 5.0% CO_2_ at 37 °C. In a 96-well plate, the cells were seeded at 2.5 × 10^4^ cells/well. After 72 h, the cells were confluent, the media was changed, and then the cells were incubated with various concentrations (300, 100, 50, 10, and 1 µM), as well as lower concentrations (500, 100, and 50 nM) were used, especially for compound **2e** against the B16-F1 cancer cell line to calculate accurate IC_50_ values for the evaluated compounds (2a–2f) for 24 h. The viability of cells was assessed by the Cell Tilter 96® Aqueous One Solution Cell Proliferation (MTS) Assay according to the manufacturer’s procedures (Promega Corporation, Madison, WI). However, at the end of the treatment, about 20 μl/100 μl of MTS solution/media was added to each well and for 2 h, they were incubated at 37 °C. Finally, the absorbance was measured at 490 nm [53].

### Statistical analysis

All of the obtained results were expressed as mean ± SD standard deviation; the result was considered significant when the *p-*value was < 0.05.

## Results and discussion

### Chemistry

**Scheme 1:**
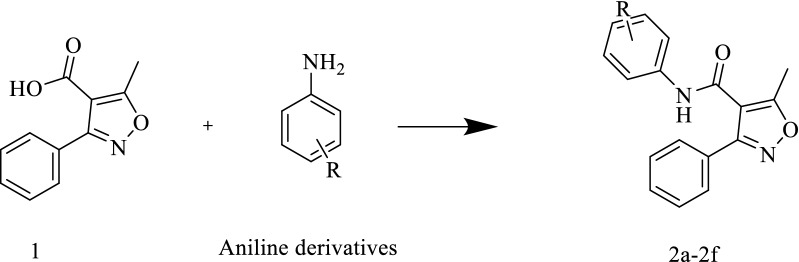
**1** + aniline derivatives stirred in 20 ml of DCM, then DMAP and EDC were added under inert gas and stirred for 48–72 h

**Scheme 2:**
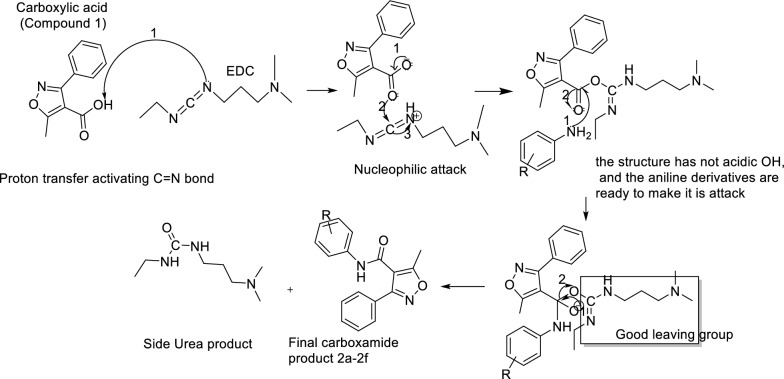
Compound **1** was deprotonated by the EDC in the first step, in the second step a nucleophilic attack was done, after that no acidic proton is present in the intermediate, and this makes the aniline derivatives able to make it is attacked easily, in the fourth step carbodiimide is leaving as good leaving group, and finally two compounds are observed the desired product (**2a–2f**) and the urea side product

The synthesis of novel 3-methyl-4-phenyl-isoxazole–Carboxamide derivatives (2a–2f) was presented in “Scheme 1”. The coupling reaction to form the 3-methyl-4-phenyl-isoxazole–Carboxamide compounds (**2a**–**2f**) was afforded by using DMAP and EDCI as activating agents and covalent nucleophilic catalysts, respectively. After 30 min, the afforded aniline derivative was added for each reaction [54], the mechanism of this coupling reaction by using the EDC as activating agent is discussed in Scheme 2 [55, 56]. Then each reaction product was purified by column chromatography using different solvent systems (*n*-hexane, ethyl acetate, DCM, and ethyl acetate). The synthesis of these derivatives was confirmed by HRMS and all product masses matched the calculated masses. The yields of all final compounds were in the range of 59–81%, and these results yields seem very close to the previous literature with similar compounds and methods [18, 21, 37, 49]. The observed signals of ^1^H-NMR spectrums of the final products showed: firstly, a singlet signal for the proton of amide in ppm range of 9.20–10.78 for all compounds, secondly multiple signals in the aromatic area were observed, then a singlet signal integrated for 3 protons was observed around 2.62 ppm, that should be related to the CH_3_ group of Isoxazole ring. Moreover, the main observed signals of ^13^C-NMR spectrum showed a clear signal around 170 ppm that should belong to carbonyl carbone, various signals were observed between 160 and 90 ppm for aromatic carbons as well signal around 12.4 ppm for aliphatic carbon CH_3_ of isoxazole ring.

### RO5 and chemoinformatics characteristics of the newly synthesized compounds (2a–2f)

The chemo-informatic properties were predicted by using computational tools. Results indicate that the newly synthesized products 2a–2f have good predicted values regarding the polar surface area (PSA; A2), molecular weight (g/mol), partition coefficient (log P), hydrogen bond donor, and acceptor (HBD and HBA, respectively). Furthermore, the RO5 tests revealed that almost all of the newly synthesized compounds, 2a–2f, follow this rule and have excellent values when compared to the standard (Table 1) [57]. The synthesized products were shown to have good oral bioavailability because all predicted data were within the reference range. Nevertheless, the drug score was calculated and used to assess the synthesized products according to electronic distribution, hydrogen bonding characteristics, flexibility, molecule size, and hydrophobicity, and depending on the results listed in Table 1, showed that most of the synthesized products have good drug scores (0.31–0.61) that define good drug-likeness behavior and may be considered as drug candidate agents versus their targets, except one product (2b) showed a bad drug score (− 0.04), which indicates that it does not have good drug-likeness behavior.

**Table 1:** Chemo-informatics characteristic of the newly synthesized compounds **2a**–**2f**

Properties	**2a**	**2b**	**2c**	**2d**	**2e**	**2f**	Standard
M.Wt. (g/mol)	372.09	346.09	400.14	324.09	362.09	360.09	< 500
HBA	5	3	5	4	4	4	< 10
HBD	1	1	1	1	1	1	< 5
Log P	3.70	4.22	4.74	3.78	4.41	4.53	< 5
PSA (A^2^)	59.85	45.29	59.75	45.29	51.23	46.04	< 89
nrotb	5	4	6	4	5	4	< 10
Drug Score	0.31	− 0.04	0.50	0.38	0.45	0.61	0.0–2.0

### Potent compound nano-emulgel

**Fig. 2:**
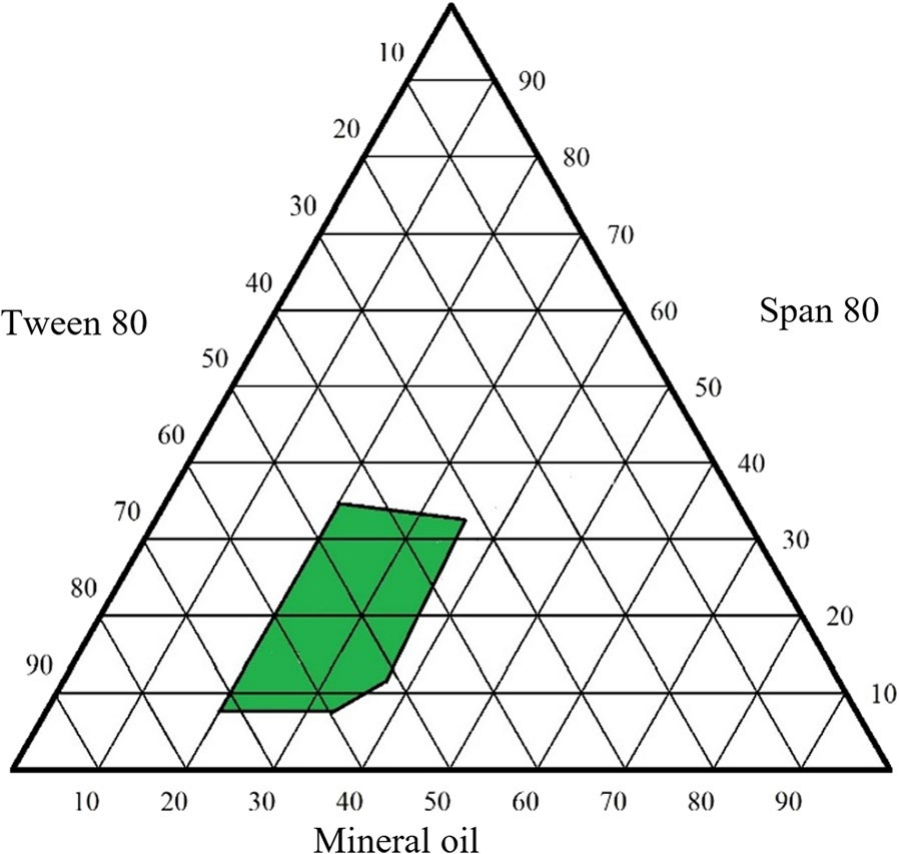
Pseudo-ternary phase diagram of mineral oil self-nanoemulsifying system

Mineral oil nano-emulsion was prepared using a self-emulsifying process. The optimal nanoemulsion was identified for the formulation based on the droplet size and polydispersity index of the mineral oil. As a result, a ternary phase diagram was produced using various quantities of mineral oil, Tween 80, and Span 80 to determine the optimal composition. As shown in Fig. 2, the ternary phase diagram exhibited a significant nano-emulsion zone. Nano-emulsion formulations with droplet size less than 200 nm were chosen. Therefore, the optimum nano-emulsion formulation (35% mineral oil, Tween 80, 50%, and Span 80, 15%) showed the lowest droplet size (70.51 ± 0.5 nm) and polydispersity index (less than 0.3). As a result, this formulation was loaded with the potent compound and showed a particle size of 74.28 ± 0.8 nm and a polydispersity index of 0.278 ± 0.7.

The small nanoparticles produced were due to the good oil solubility of the potent compound as it is composed of fatty acids, which helps in developing a formulation with nanoscale particles. In addition to that, the used surfactants were safe, non-ionic (hydrophilic), and biocompatible. By making the surface area large as possible, rapid drug release and absorption were conducted [58]. Moreover, the use of Tween 80 and Span 80 was able to give good emulsification properties to the mineral oil, which helps in producing the nanoparticles [51, 59].

The polydispersity index is critical in determining the stability of the nano-emulgel formulation since it represents the population size distribution within a specific sample. When the polydispersity index is large, the homogeneity of the particles in the formulation decreases [58]. The polydispersity index of the nano-emulgel formulation is less than 0.3, indicating a narrow and homogeneous globule size distribution and classifying it as a high-stable formulation. A low polydispersity index value indicates a stable nano-emulgel in this investigation [60, 61].

The nano-emulgel stability is also related to its zeta potential. The large negative and positive zeta potential values create a repulsive force between particles, which stabilizes the dispersion. Otherwise, when the zeta potential is low, the dispersion is unstable, which means there is no force keeping the particles apart. Generally, a value of 30 mV or − 30 mV is used to denote the stability of dispersions, with values more than 30 mV and less than − 30 mV being considered stable systems [59, 62]. As seen in the data, the nano-emulgel had a − 36 ± 1.3 mV value due to the non-ionic surfactants included in the formulation, which covered the system surrounding the surface, assisting in its stabilization. In comparison to particles, they did not affect the stability of the nano-emulsion [63].

## Biological evaluations

### Cytotoxic evaluation of the compounds 2a–2f

To evaluate the antiproliferative activities of the synthesized compounds, the MTS assay was performed on B16-F1, Colo205, HepG2, Hep3B, CaCo-2, HeLa, and MCF7 cells, as well as the normal cell line, Hek293t. As shown in Table 2, seven concentrations were used (300, 100, 50, 10, 1, 0.5, 0.1 and 0.05 µM). Based on the results shown in Table 2, the compound 2a showed a broad range of activities on five cancer cell lines with an IC_50_ range of 7.54–129.17 µM, and this compound was the most potent structure against Colo205 and HepG2 cancer cell lines, with IC_50_ values of 9.179 and 7.55 µM, respectively, as well as potent on normal cell lines Hek293t with an IC_50_ of 2.54 µM. All of the synthesized compounds (**2a–2f**) showed potent to moderate activities against B16F1 with an IC_50_ range of 0.079–42.93 µM and the most active compound was the 2e compound. In contrast, our synthesized compounds showed weak or negligible activities against Hep3B, CaCo-2, HeLa, and MCF7 cancer cell lines.

**Table 2:** IC_50_ (µM) of phenyl-isoxazole–Carboxamide compounds (**2a–2f**) on various cell lines


Code	**2a**	**2b**	**2c**	**2d**	**2e**	**2f**
R	2,5–OMe 4–Cl	3–CF_3_	4-(2-methoxy phenoxy)	4-SCH_3_	4–OCF3	4-(thiophen-2-yl)
	IC_50_ µM					
Cell line	**2a**	**2b**	**2c**	**2d**	**2e**	**2f**
B16F1	29.72 ± 2.12	42.93 ± 1.55	21.13 ± 0.78	39.84 ± 1.45	**0.079** ± 0.004	**12.72** ± 1.01
Colo205	**9.18** ± 0.88	> 200	183.45 ± 2.05	216.38 ± 1.58	> 200	75.40 ± 2.41
HepG2	**7.55** ± 0.79	53.58 ± 2.04	120.02 ± 2.19	37.86 ± 2.07	> 200	38.38 ± 1.07
CaCo-2	129.17 ± 2.47	> 200	> 200	> 200	> 200	> 200
HeLa	40.84 ± 1.25	> 200	> 200	> 200	> 200	> 200
Hep3B	> 200	> 200	> 200	> 200	> 200	54.61 ± 2.11
MCF-7	> 200	> 200	> 200	> 200	> 200	> 200
Hek293t	2.54 ± 1.40	147.58 ± 2.74	20.18 ± 1.32	> 200	190.16 ± 2.67	7.91 ± 0.61

*p* value ≤ 0.05

**Fig. 3:**
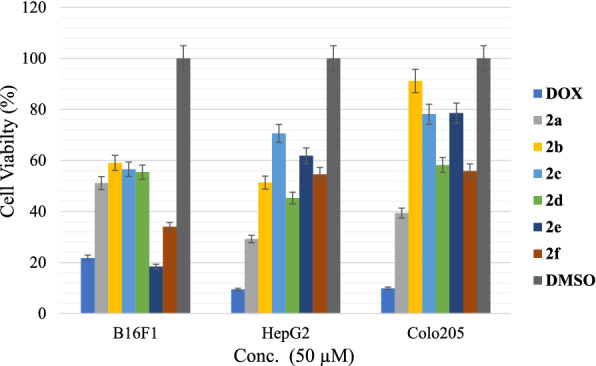
Cell viability percentages against B16F1, HepG2, and Colo205 for all synthesized compounds versus Dox (positive control) and DMSO (negative control)

Cell viability percentage was calculated for B16F1, Colo205, and HepG2 cancer cells at 50 µM concentrations and compared to positive control doxorubicin (Dox) and negative control DMSO, as shown in Fig. 3. The viability percentage for 2a–2d compounds against B16F1 was around 55%, while the percentages were 18.42% and 34.03% for compounds **2e** and **2f** respectively, in comparison with the dox percentage, which was 21.78%, which means that compound **2e** has more activity against B16F1 in comparison with the positive control (Dox). The cell viability percentage for compound **2a** against HepG2 and Colo205 was 29.24% and 39.39% respectively, in comparison with Dox, values of 9.46% and 9.89%.

**Fig. 4:**
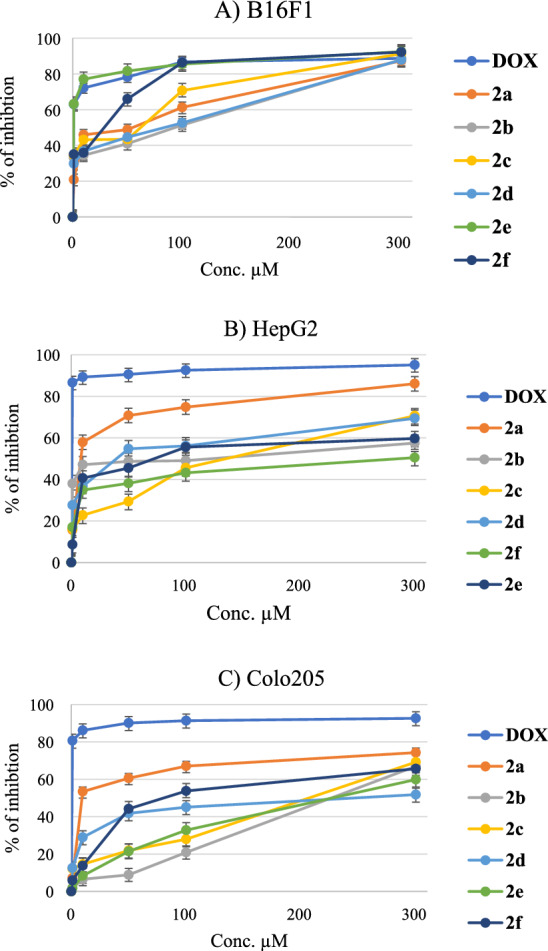
The percentage of inhibition of selected cancer cell lines (A: B16F1; B: HepG2; C: Colo 205) after treatment with the synthesized compounds (**2a–2f**) and positive control (Dox)

The cell index inhibition percentage was calculated regarding five concentrations (300, 100, 50, 10, and 1 µM) for the selected cancer cell lines (B16F1, HepG2, and Colo205), and presented in Fig. 4. It was clear that the inhibition percentage of compound 2e against the B16F1 (Fig. 4A) cancer cell line was very close to the percentage of inhibition of positive control (Dox), and the closest compound against HepG2 and Colo205 to the inhibition percentage of Dox was compound 2a (shown in Fig. 4B, C).

### SAR analysis

The isoxazole heterocycle has very important pharmacological activities, especially as anticancer agent according to the recent publications on this core cycle [64], this fact is confirmed as the isoxazole heterocycle could be one of the main pharmacophores for antiproliferative activities, on the other side the activities were better against Hep3B or MCF-7 when the 3-phenyl ring was substituted with halogen (F and/or Cl) [18, 21, 37, 49], while in this study the 3-phenyl ring was unsubstituted and there were very weak activities against these cancer cell lines. Moreover, the substituted groups like CF_3_ or OCF_3_ on phenyl ring in recent works produced potent anticancer agents [65, 66], especially against melanoma cancer cell line HS27, and this can be confirmed that the most active compound (**2e**) against Melanoma cancer cell line (B16F1) was substituted with OCF_3_ group, this group was considered as one of the main pharmacophore sections for anticancer agents [67, 68], as well as when N-phenyl is substituted at para position with t-butyl substitution showed the most potent activities on cancer cell lines, and this observation was observed previously on our previous works [21, 49].

## Nano-enulgel results of compound 2e

**Fig. 5:**
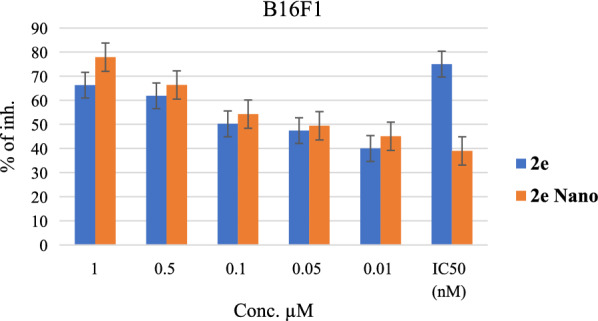
: The percentage of inhibtion of compound **2e** versus **2e** nano at 1, 0.5, 0.1, 0.05, and 0.01 µM concentrations and their IC_50_ values

The most active compound against the B16F1 cancer cell line was the **2e** compound with an IC_50_ of 79 nM, and to increase its potency against this cancer cell line, nano-emulgel of this compound was used at 1, 0.5, 0.1, 0.05, and 0.01 µM concentrations and compared the standard compound without nanoemulgel. The results showed that the IC_50_ values of nano-emulgel for the 2e compound were decreased to 39 nM in comparison with the standard 2e compound, which was 79 nM. This means that the nanoemulgel increased the potency of this compound fold more than the standard compound. Figure 5 showed the increase of the percentage of inhibition in all used concentrations. This result is attributed to the high penetration of the potent compound nano-emulgel, which is facilitated by the nanoparticles' tiny size and wide surface area, which enhances the nano-emulgel's interaction with cancer cell lines [69]. Moreover, nano-emulgel increased the intracellular uptake of the anticancer agent, which led to an increase in its therapeutic activity. In addition to that, the increase in intracellular retention also plays an important role in increasing efficacy [70].

### Anti-fibrotic activity

**Fig. 6:**
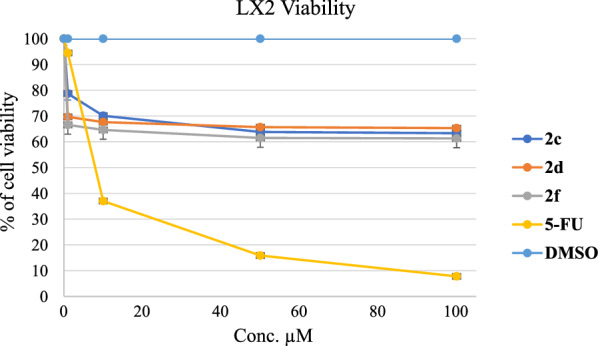
The cell viability of LX2 cell line after treatment with the synthesized compounds **2a–2f** and positive control **5-FU**

To explore the anti-fibrotic effects of these compounds on human hepatic stellate cell (HSC) line LX-2, the viability of LX-2 cells following various compounds' treatment was determined by the MTS assay. All compounds showed moderate antifibrotic activities. The most active compounds were selected and presented in Fig. 6. They showed very similar activities. The most potent was compound 2f, and the cellular viability of LX-2 was 66.55% at 1 μM concentration in comparison with the positive control 5-FU cell viability value of 94.64%. The results suggested that these compounds have better antifibrotic activities than 5-FU at 1 μM concentration, and further biological investigation into the LX2 cell line is requested soon.

## Conclusion

The synthesized compounds **2a–2f** showed different activities on B16-F1, Colo205, HepG2, Hep3B, CaCo-2, HeLa, and MCF7 cancer cell lines, ranging from moderate to potent activity compared with 5-FU and Dox anticancer drugs. The most potent compound, **2e,** shows great activity on the B16-F1 cancer cell line, with an IC_50_ value very close to the Dox value (IC_50_ = 0.079 and 0.056 μM, respectively). To increase its activity on this cancer cell, a nano-emulgel was prepared to gain a fold effect, which improved from 79 to 39 nM in the nano form. The antifibrotic activities of the synthesized compounds at low concentrations were better than those of 5-FU. The synthesized compounds, especially in the nano form, could be a promising agent for melanoma cancer and further in vitro and in vivo studies should be conducted in the future.

## Supplementary Information

**Additional file 1.** contain the IUPAC name, chemical structures and NMR spectrums of 2a-2f compounds.

## Data Availability

All data generated or analysed during this study are included in this published article.

## Abbreviations

ECM: Extracellular matrix
HSC: Hepatic stellate cells
NMR: Nuclear magnetic resonance
M.P.: Melting points
HRMS: High-resolution mass spectrometer
DMSO: Dimethyl sulfoxide
IC_50_: Half maximal inhibitory concentration
MCF7: Human breast cancer cell line
HeLa: Human cervix adenocarcinoma cell line
Dox: Doxorubicin
Conc.: Concentration
