# Supplementary material for: Synthesis of novel isoxazole–carboxamide derivatives as promising agents for melanoma and targeted nano-emulgel conjugate for improved cellular permeability
Source: BMC Chem. 2022 Jun 24;16(1):47. doi: 10.1186/s13065-022-00839-5 (PMC9229817; doi:10.1186/s13065-022-00839-5)

**Synthesis of novel isoxazole-carboxamide derivatives as promising agents for melanoma and targeted nano-emulgel conjugate for improved cellular permeability.**

Mohammed Hawash^a^*, Nidal Jaradat^a^, Ahmad M Eid^a^, Ahmad Abubaker^a^, Ola Mufleh^a^, Qusay Al-Hroub^a^ and Shorooq Sobuh^b^

^1^Department of Pharmacy, Faculty of Medicine and Health Sciences, An-Najah National University, Nablus P.O. Box 7, 00970, Palestine.

^2^Department of Biomedical Sciences, Physiology, Pharmacology & Toxicology Division. Faculty of Medicine and Health Sciences, An-Najah National University, Nablus, Palestine

Corresponding author:

Mohammed Hawash, **E-mail:* [*mohawash@najah.edu*](mailto:mohawash@najah.edu)*, +972-569-939-939.*

ORCID No. 0000-0001-5640-9700

Running title: Nanoformulation of Novel Isoxazole derivatives as promising agents for Melanoma

**OQA 5 (2a)**

**Chemical formula:** C_19_H_17_ClN_2_O_4_

**IUBAC Name: -**N-(4-chloro-2,5-dimethoxyphenyl)-5-methyl-3-phenylisoxazole-4-carboxamide


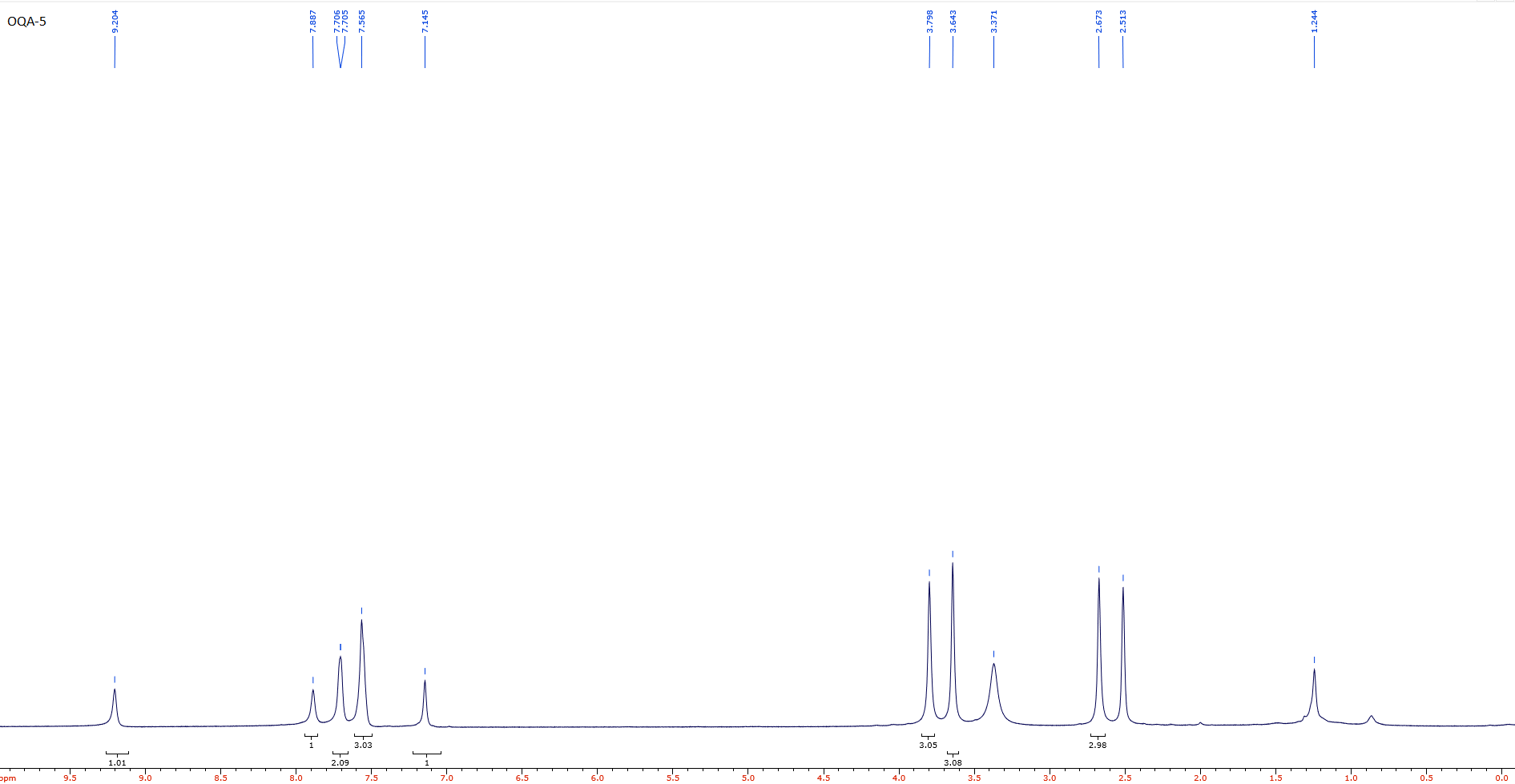


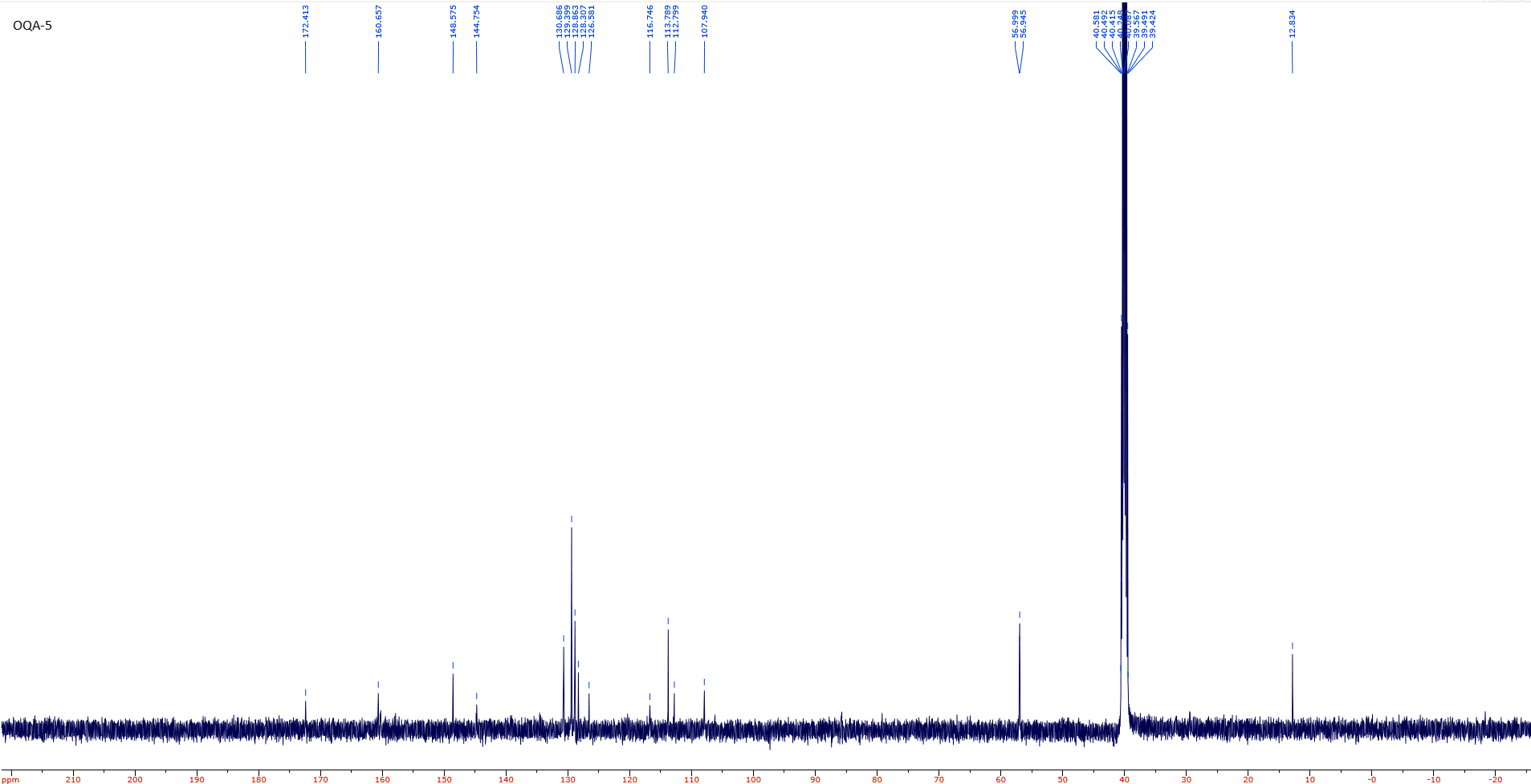


**OQA 6 (2b)**

**Chemical formula:** C_18_H_13_F_3_N_2_O_2_

**IUBAC Name:** 5-methyl-3-phenyl-N-(3-(trifluoromethyl) phenyl) isoxazole-4-carboxamide

**
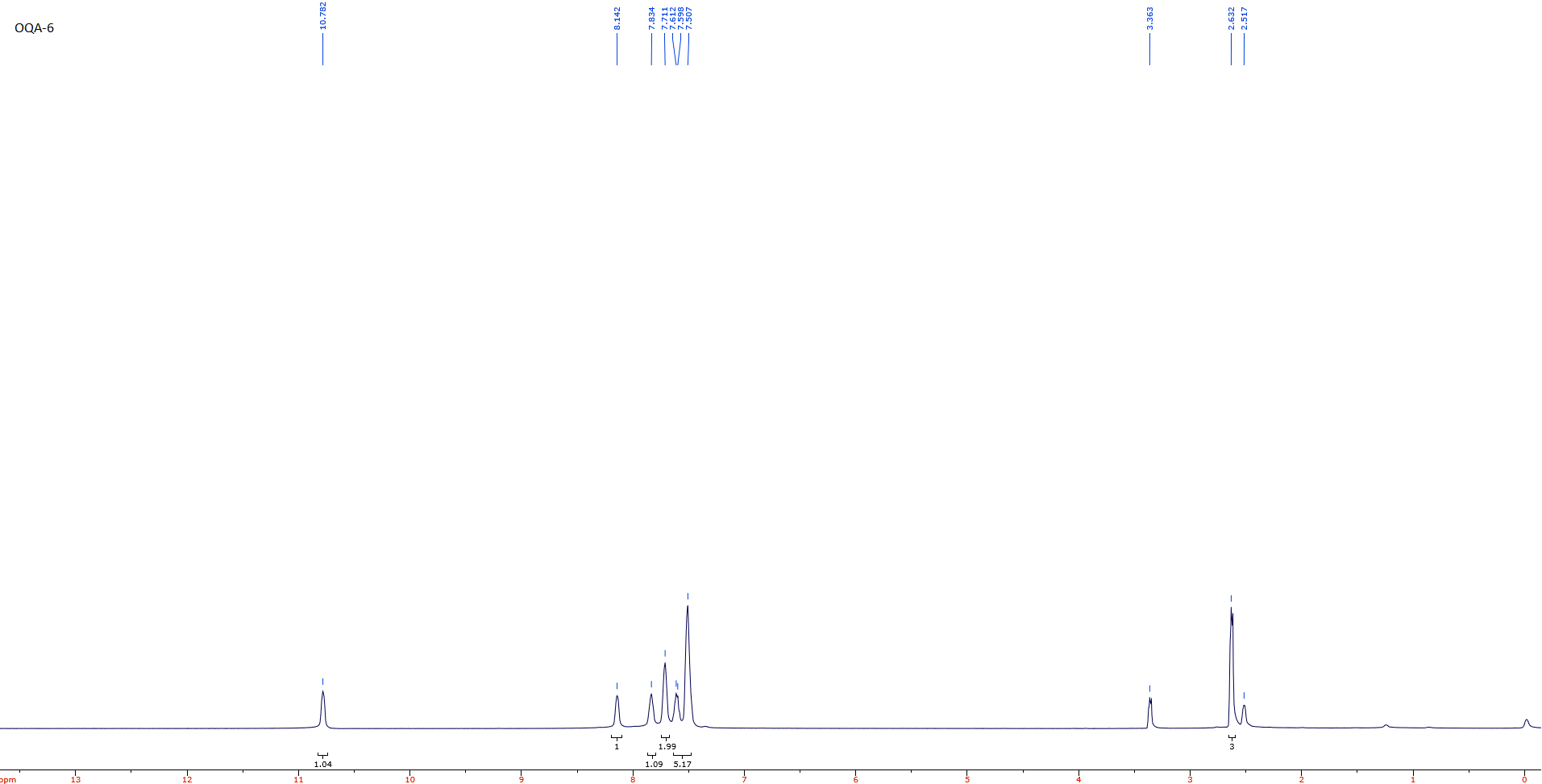

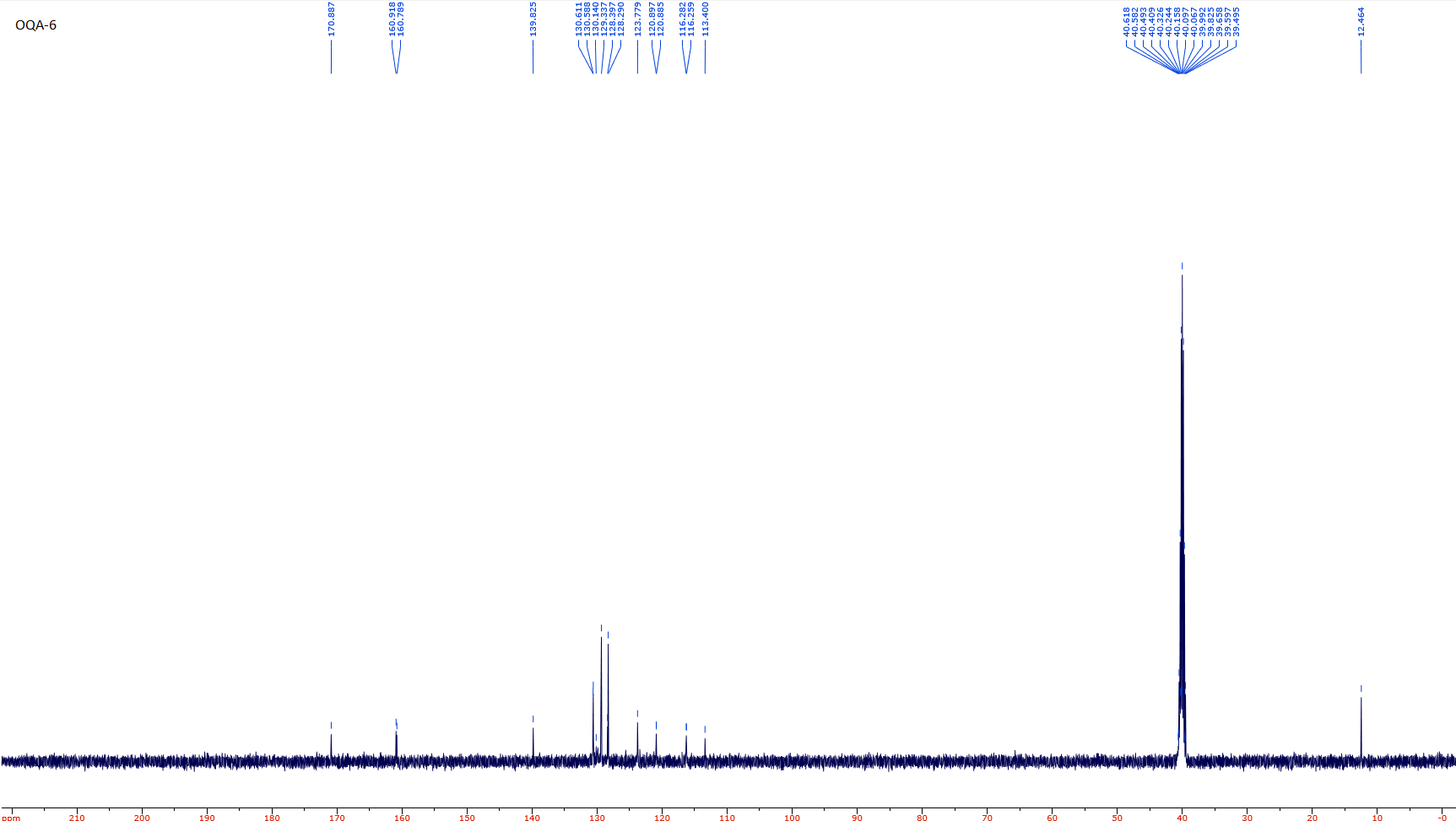
**

**OQA 7 (2c)**

**Chemical formula:** C_24_H_20_N_2_O_4_

**IUBAC:** N-(4-(2-methoxyphenoxy) phenyl)-5-methyl-3-phenylisoxazole-4-carboxamide

**
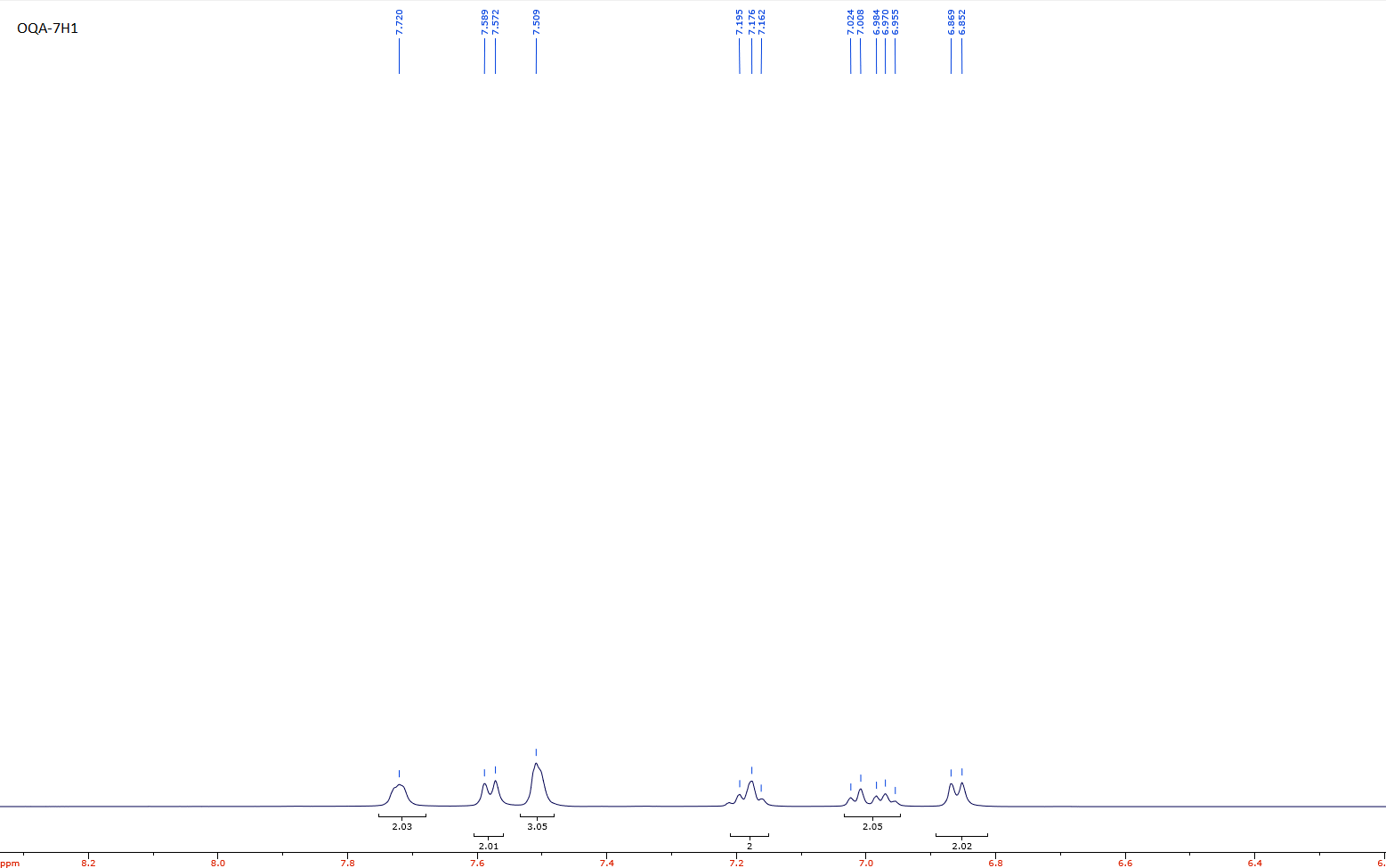
**

**
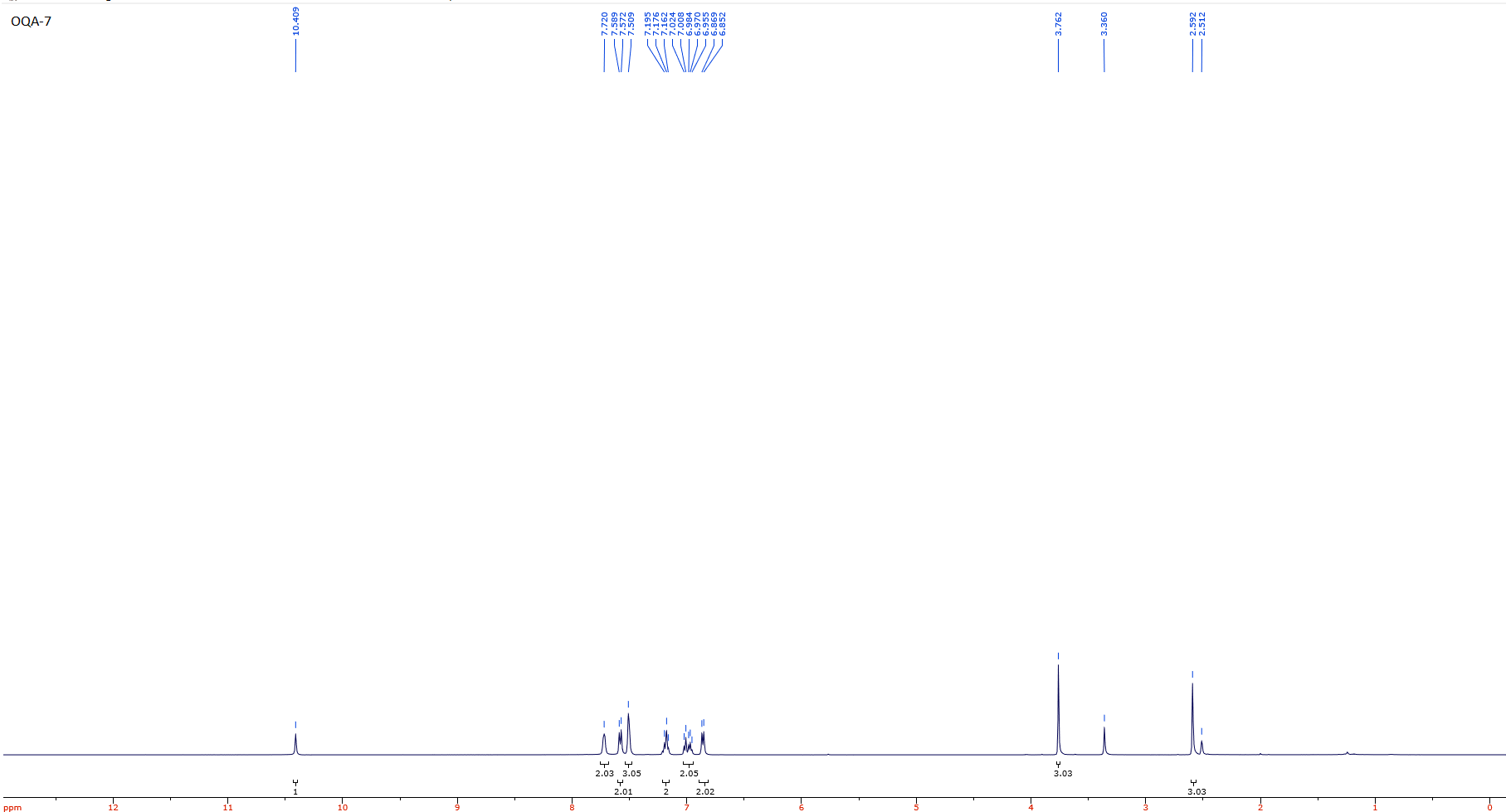
**

**
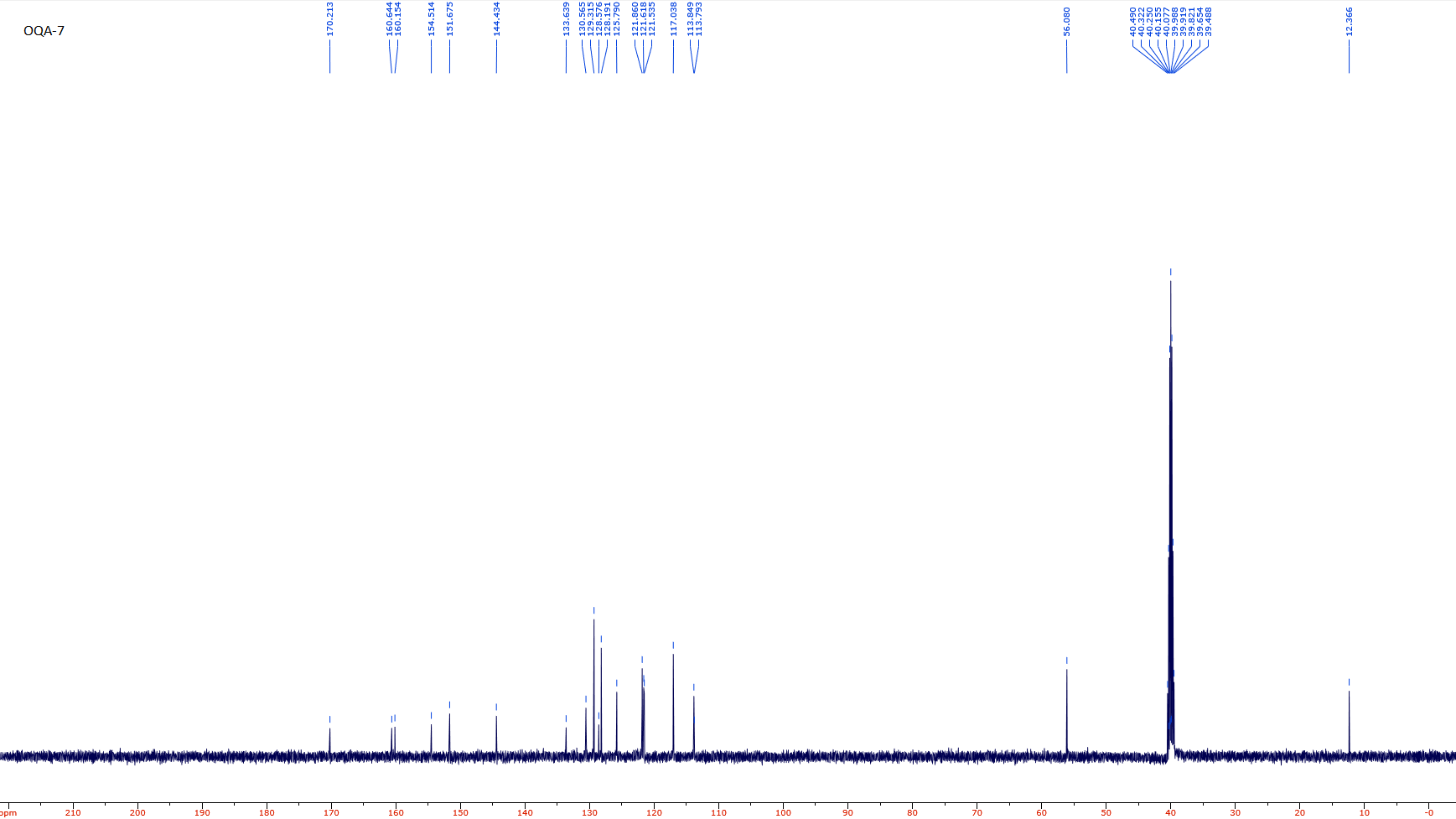
**

**OQA 8 (2d)**

**Chemical formula:** C_18_H_16_N_2_O_2_S

**IUBAC Name:** 5-methyl-N-(4-(methylthio) phenyl)-3-phenylisoxazole-4-carboxamide


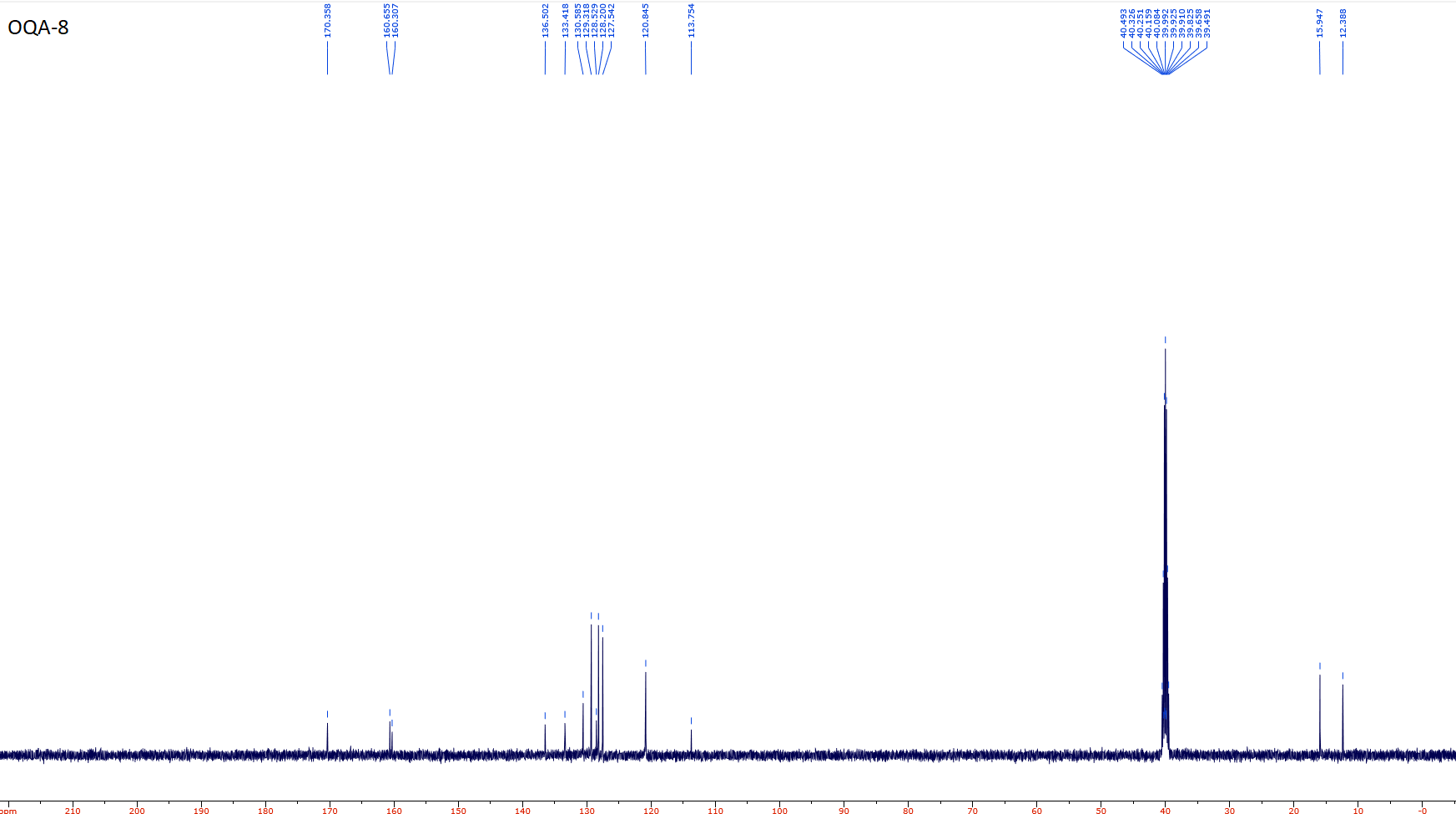

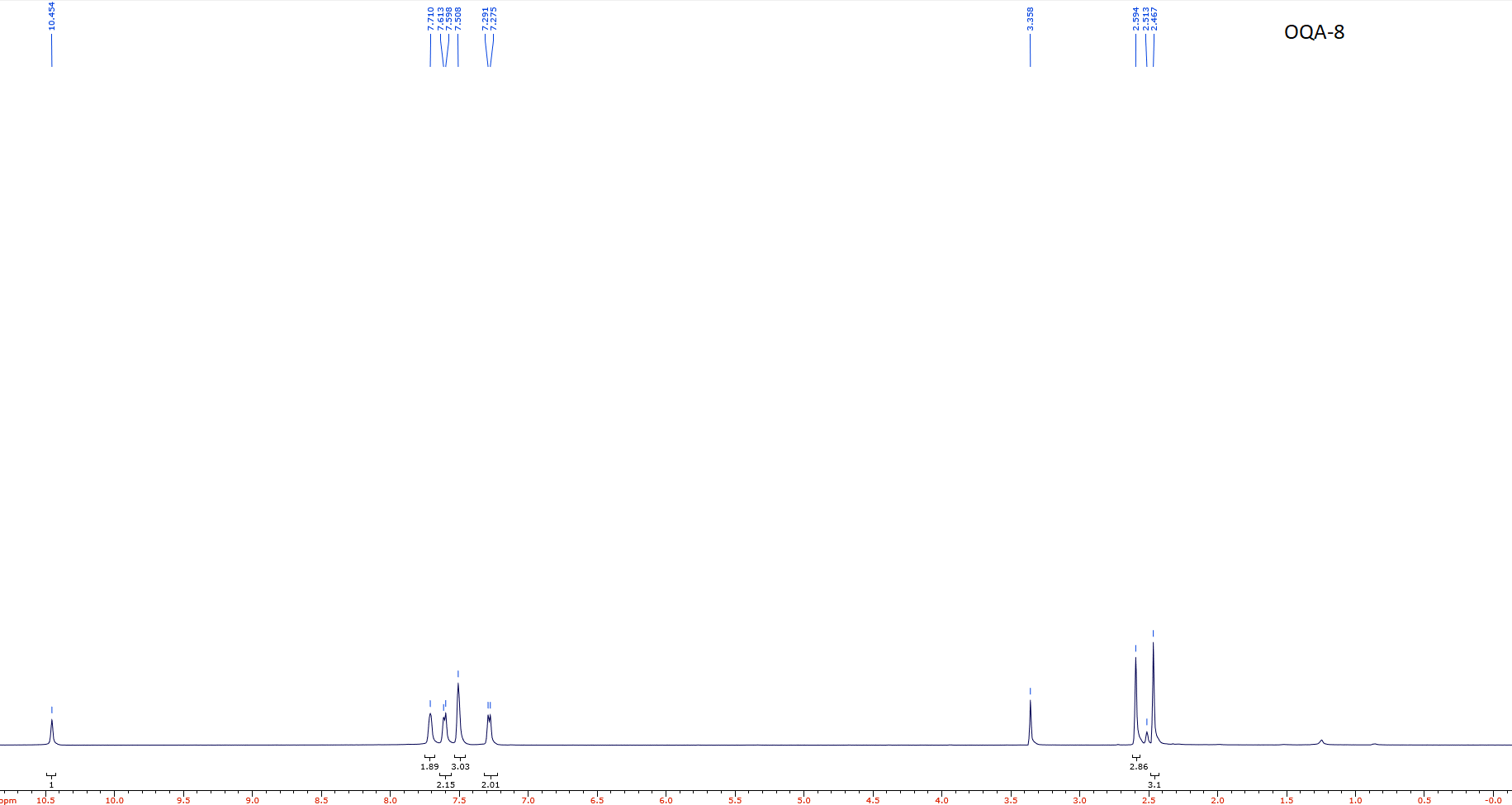


**OQA 10 (2e)**

**Chemical formula:** C_18_H_13_F_3_N_2_O_3_

**IUBAC:** 5-methyl-3-phenyl-N-(4-(trifluoromethoxy) phenyl) isoxazole-4-carboxamide


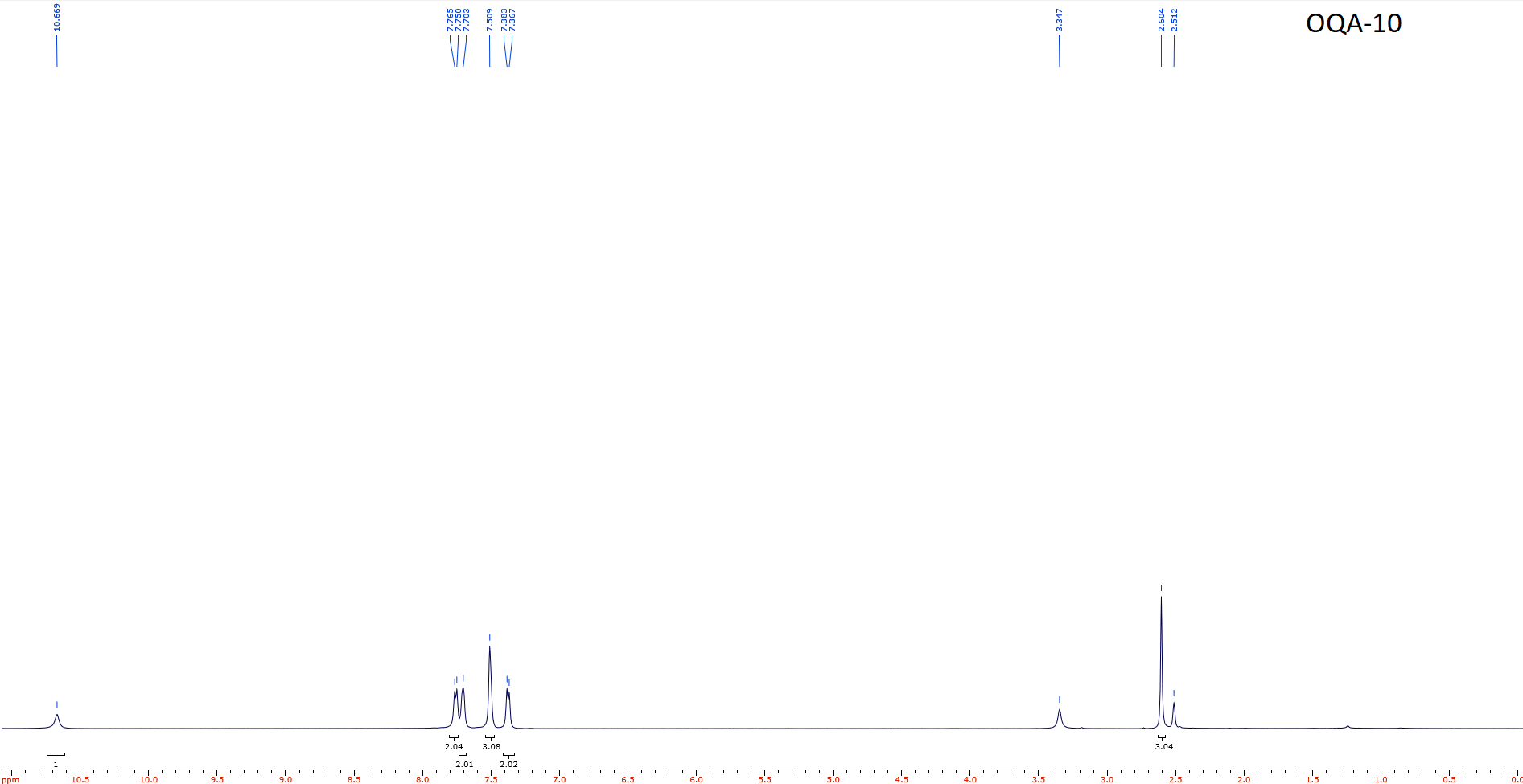


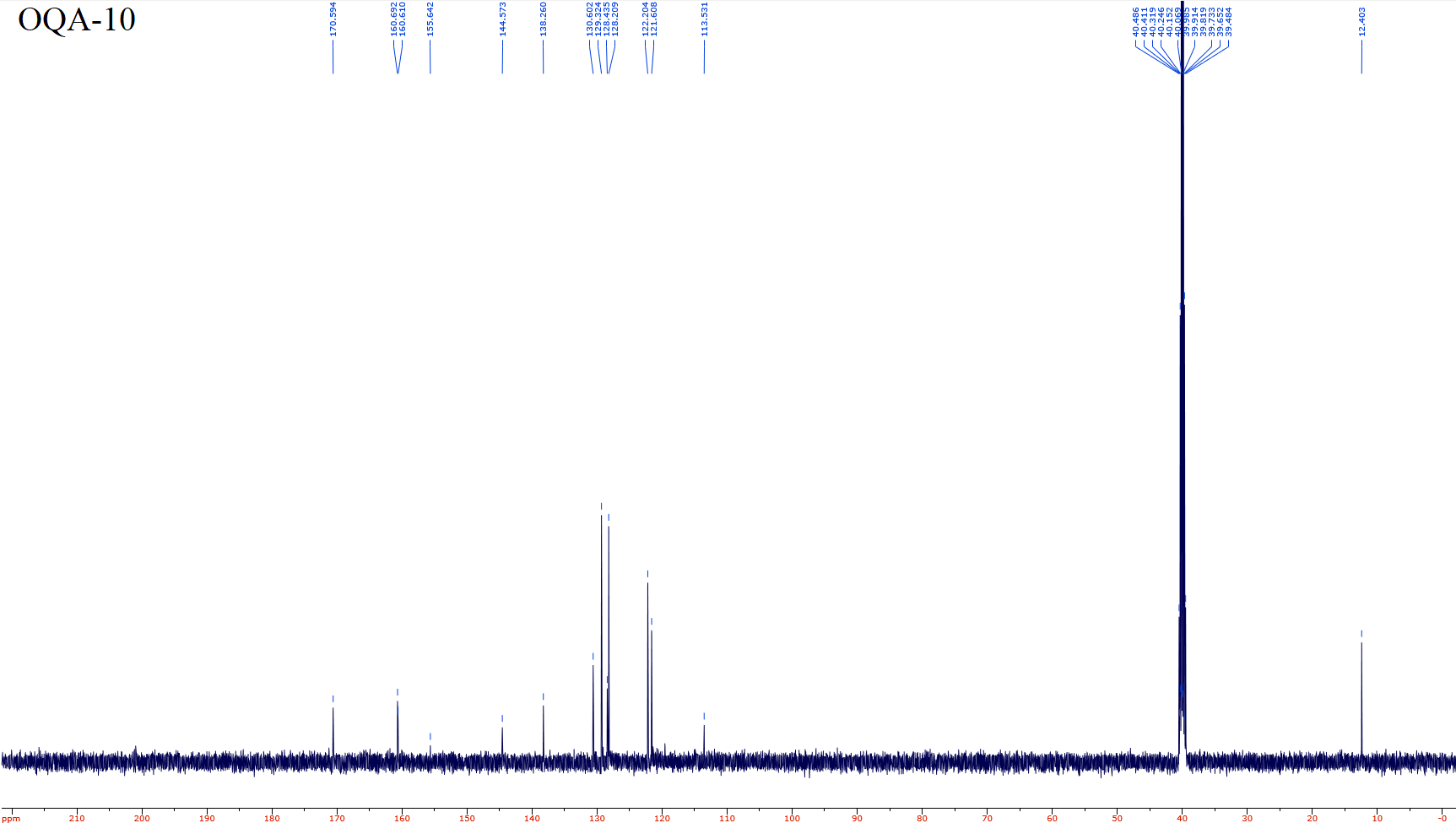


**OQA 14 (2f)**

**Chemical formula:** C_21_H_16_N_2_O_2_S

**IUBAC:** 5-methyl-3-phenyl-N-(4-(thiophen-2-yl) phenyl) isoxazole-4-carboxamide


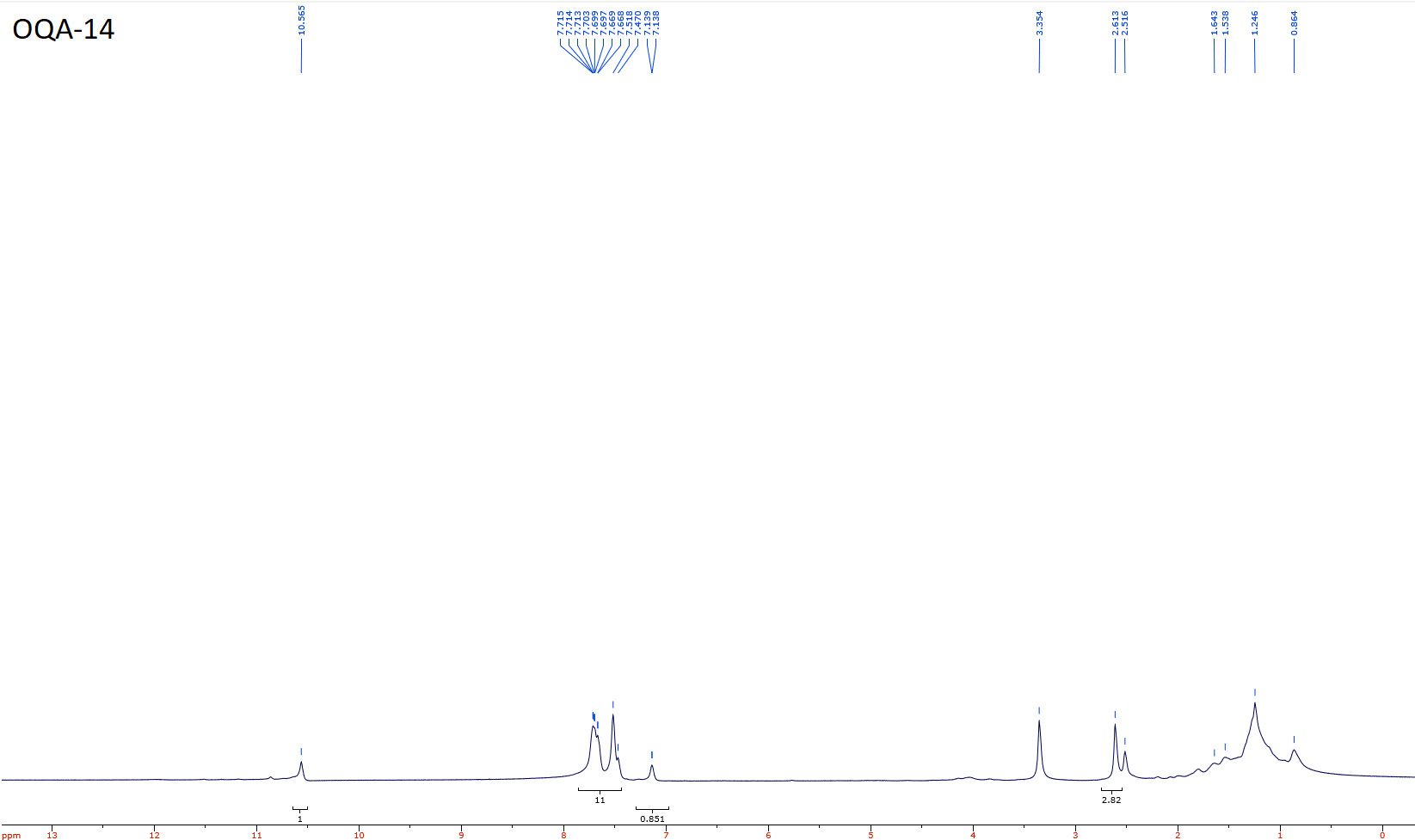


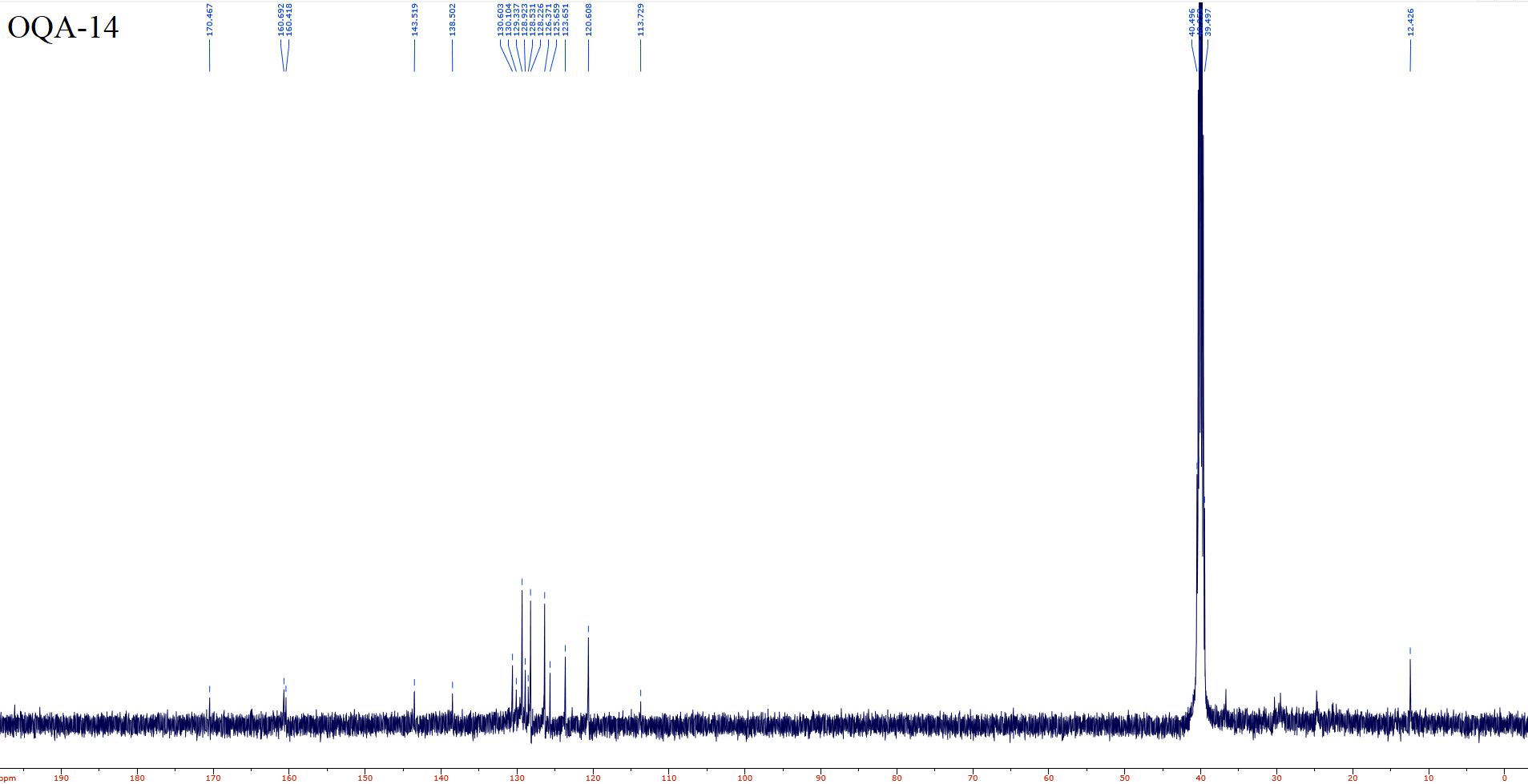


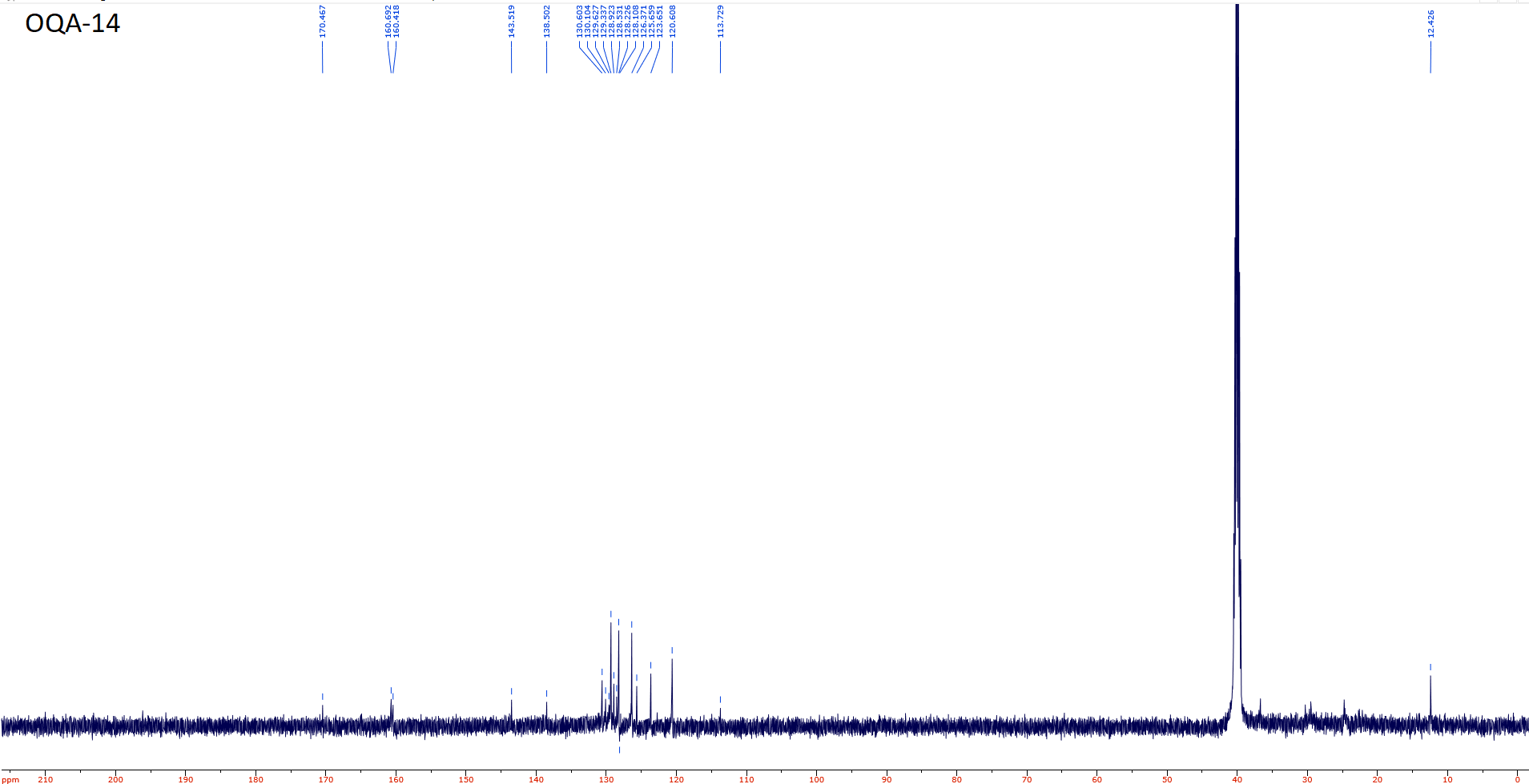

Supplement: Supplementary file 1 — Additional file 1. contain the IUPAC name, chemical structures and NMR spectrums of 2a-2f compounds. [file 13065_2022_839_MOESM1_ESM.doc]
